# Choroidal structure as a biomarker for visual acuity in intravitreal aflibercept therapy for polypoidal choroidal vasculopathy

**DOI:** 10.1371/journal.pone.0197042

**Published:** 2018-05-10

**Authors:** Shotaro Asano, Keiko Azuma, Kimiko Shimizu, Risako Yamamoto, Jinhee Lee, Hiroshi Murata, Tatsuya Inoue, Ryo Asaoka, Ryo Obata

**Affiliations:** 1 Department of Ophthalmology, Graduate School of Medicine and Faculty of Medicine, The University of Tokyo, Tokyo, Japan; 2 Miyata Eye Hospital, Miyazaki, Japan; Massachusetts Eye & Ear Infirmary, Harvard Medical School, UNITED STATES

## Abstract

**Purpose:**

To investigate the relationship between choroidal structure and visual acuity after intravitreal aflibercept therapy for polypoidal choroidal vasculopathy (PCV).

**Methods:**

We conducted a retrospective, single-centre and observational study including 18 eyes of 18 patients with PCV (73.8 ± 10.2 years of age) who were treated with three monthly intravitreal aflibercept injections followed by additional treatments in a treat-and-extend protocol. The cross-sectional images of the macula were obtained with enhanced depth imaging optical coherence tomography at baseline, at 3 months, and at 12 months. The choroidal layer was divided into luminal or stromal segments by applying binarization processing to calculate these areas. The relationships between age, spherical equivalent, best-corrected visual acuity (BCVA), baseline value, or changes in the luminal or the stromal areas, and the BCVA change at 12 months were analysed using multiple regression analyses and model selection procedures.

**Results:**

Both stromal and luminal areas were decreased at 3 and 12 months compared to baseline areas (5% and 9% at 3 months, 6% and 12% at 12 months, p < 0.0001, p < 0.0001, p < 0.0001 and p < 0.0001, respectively). Greater improvement of visual acuity (VA) at 12 months was significantly associated with younger age, greater spherical equivalent, worse baseline BCVA, greater baseline luminal area, and smaller baseline stromal area.

**Conclusions:**

Choroidal structure might be useful as a new biomarker for potential Visual outcomes after intravitreal aflibercept therapy for PCV.

## Introduction

Exudative age-related macular degeneration (AMD) is a leading cause of blindness globally [1]. Polypoidal choroidal vasculopathy (PCV) is considered as a subtype of exudative AMD, and is characterized by polypoidal dilation of abnormal vessels beneath the retinal pigment epithelium (RPE), with connecting vascular networks [2]. PCV is more commonly seen in Asian compared with Caucasian populations [3].

Studies have demonstrated that intravitreal injections of anti-vascular endothelial growth factor (VEGF) drugs such as ranibizumab, bevacizumab, and aflibercept are effective for PCV as well as for typical exudative AMD [4–6]. Because the response to therapy is different among patients, the treatment intervals are modified in most cases based on the patient’s response [7–9].

Recent advances in retinal imaging, such as the use of optical coherence tomography (OCT), have revealed that the anatomical findings in the choroid are significantly associated with the pathogenesis of and the treatment efficacy for PCV. PCV shows a greater choroidal thickness than typical AMD [10–12], suggesting that aberrant choroidal circulation is associated with the development of PCV [13]. Studies have demonstrated that choroidal thickness significantly decreased after anti-VEGF treatment for PCV [14–16]. Of note, several studies have shown that the choroidal thickness might be significantly associated with visual outcomes [5, 17–19], but the results in these reports were controversial. A study [19] demonstrated no statistical difference in visual outcomes after the treatment for PCV. In contrast, the other studies [5, 17, 18] reported that greater choroidal thickness at baseline predicted better visual change after the treatment. Choroidal circulation is essential for the retina because oxygen and nutrition is provided from the choroid, and choroidal circulation removes metabolic waste and debris, from the outer retina and the RPE cells [20–23]. Therefore our group hypothesized that choroidal morphology could be associated with visual prognosis [24, 25]. Detailed characterization of choroidal structure in PCV can contribute to elucidate the association between choroidal changes and retinal function, and is therefore of great interest.

The choroid has two distinct components; the vascular region (lumen) and interstitial region (stroma) [23, 26]. Until recently, there have been a few studies using binarization processing to investigate how the inner structure of the choroid changes in cases with PCV using binarization processing [27, 28]. Binarization of the choroidal structure using the enhanced depth imaging (EDI)-OCT is an established method used to differentiate the luminal (vascular) choroidal area from stromal (interstitial) choroidal area [29–33]. Because previous studies suggested that the responses after anti-VEFG treatment were different between the lumen and the stroma [27, 28], the pathological changes or the responses to the treatment might be independent of each other. However, there has been no report that investigated how the luminal space or the stromal area changed after treatment and how they are individually associated with visual outcomes.

In the current study, we hypothesized that the baseline value or the change in the luminal or the stromal choroidal structure in patients with PCV could be associated differently with visual acuity after anti-VEGF therapy. We investigated the luminal and the stromal areas in the choroid at baseline and after injection of aflibercept, one of the anti-VEGF drugs, and analysed the relationship between these structures and the visual acuity at the 1-year follow-up.

## Materials and methods

### Study population

In the current study, we retrospectively reviewed the charts of patients in the outpatient clinic of the University of Tokyo Hospital. The current study was in accordance with the tenets of the Declaration of Helsinki and was approved by the institutional Review Board (IRB) of the University of Tokyo as a retrospective review of the patients’ medical records. All data were fully anonymized before we accessed them. Written informed consent was not required by the IRB but participants who did not grant authorization to use their medical records for research were excluded from analyses.

Eighteen eyes of 18 consecutive patients with PCV who had presented from December 2013 to July 2016 at the University of Tokyo Hospital, and had three monthly intravitreal aflibercept (2 mg/0.05 mL) injections (IAI) followed by additional IAI in a treat-and-extend protocol (TAE; detailed below) until 1 year, were enrolled in this study. The diagnostic criteria for PCV were: (1) a protruded orange-red coloured lesion under the RPE as observed using fundoscopy; and (2) polypoidal structure(s) on indocyanine green angiography, at the border of a branching choroidal vascular network [34]. Exclusion criteria were: (1) any previous treatments for PCV, (2) any intraocular surgery during the follow-up period, (3) a history of pars plana vitrectomy.

### Treat-and-extend (TAE) aflibercept protocol

All the patients had been treated with three monthly IAI followed by additional IAI in a TAE protocol [34, 35]. In the TAE protocol, the treatment interval was decided using funduscopic examinations with slit-lamp biomicroscopy and OCT imaging. If new hemorrhage was found on slit-lamp biomicroscopy, or fluid was observed on OCT (intraretinal and/or subretinal fluid) at the time of injection, treatment was continued at 4-week intervals. In cases when these findings continued to be absent, the next injection was extended by 2 weeks, with a maximal treatment interval of 12 weeks. When these findings of recurrence were observed at the time of any visit, the next injection was shortened by 2 weeks. Minimum intervals for the injections were 4 weeks.

### Baseline and follow-up examinations

All patients had been submitted comprehensive ophthalmic examinations including the measurement of best-corrected visual acuity (BCVA), slit-lamp biomicroscopy, funduscopy, and spectral domain OCT (Heidelberg Spectralis^®^. Heidelberg, Germany) at each visit. BCVA was measured as the decimal best-corrected visual acuity using the Landort C chart, but converted to the logarithm of the minimum angle of resolution (logMAR) visual acuity (VA). For choroidal imaging, the EDI-OCT technique was used and horizontal and vertical B scans including 768 A-scans with visual angle of 30° through the foveal centre were obtained. All images were obtained under the eye-tracking system with 100 scans averaged to minimize the speckle noise. The choroidal-scleral interface was manually delineated on an EDI-OCT image.

### Binarization of the choroid

**Fig 1:**
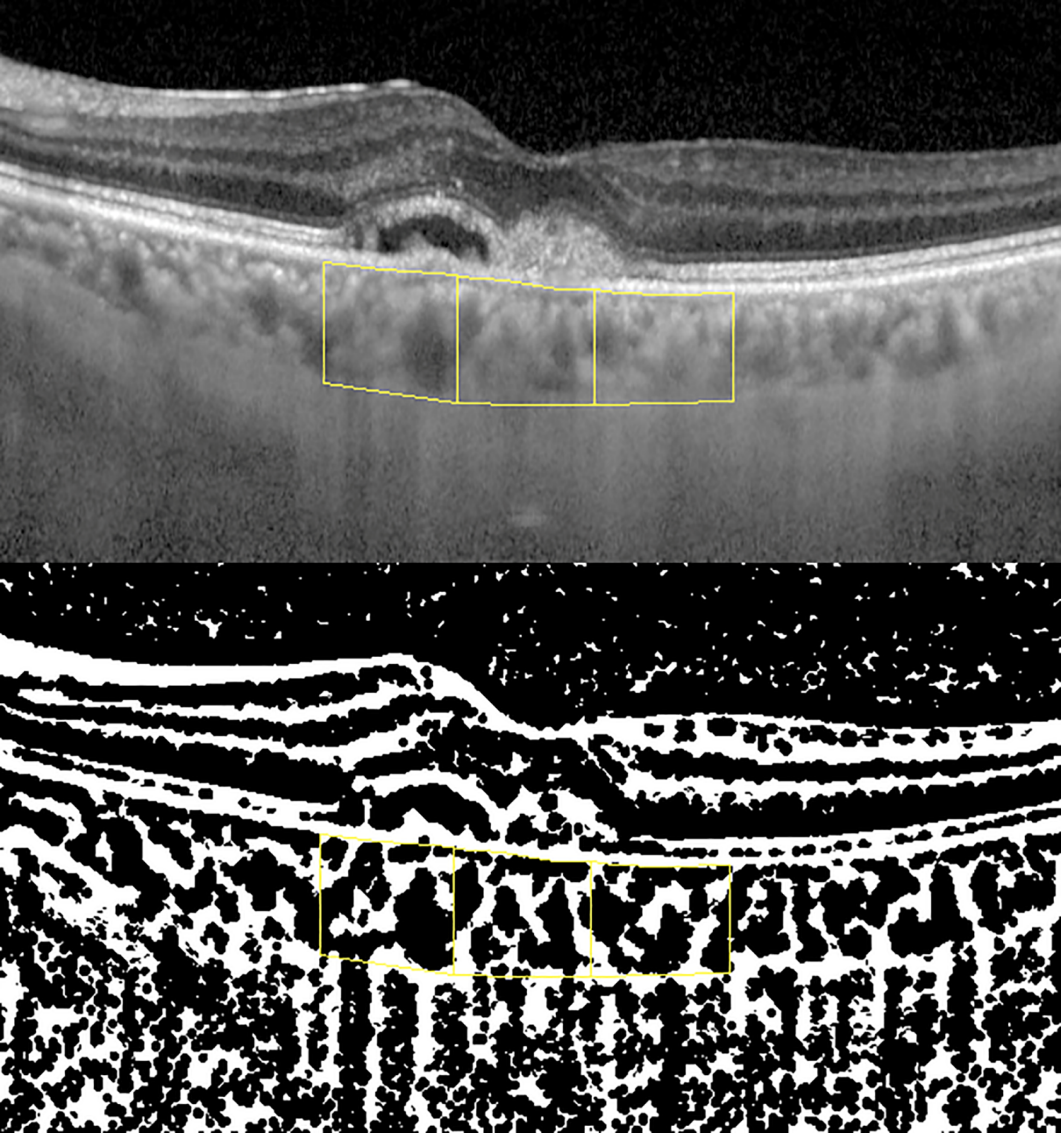
Segmentation and binarization of EDI-OCT images. (Top) From the enhanced depth imaging optical coherence tomography images, subfoveal choroidal layers under the Bruch’s membrane were divided into three sectors with each width of 1,000 μm (yellow boxes). (Buttom) The Niblack auto local threshold was applied to binarize the images to subdivide them into the stromal and luminal choroid. Then, areas were calculated using the built-in measurement tool in ImageJ.

The choroidal area with 3,000 μm of width centred on the fovea was identified in the horizontal or vertical EDI-OCT scans across the fovea. Choroidal layers under the Bruch’s membrane were then divided into three sectors with each having a width of 1,000 μm as the central, nasal, and temporal sectors, and from the horizontal scan, as the central, superior, and inferior sectors from the vertical scan. All measurements were performed by two investigators (S.A., K.A.) who were masked to all patient information. In terms of the inter-grader agreement, the intra-class correlation coefficient (ICC) between the graders was 0.97 [95 confidence interval (CI), 0.96–0.98], meaning “excellent” repeatability [36]. Using ImageJ software (version 1.51, http://imagej.nih.gov/ij/; provided in the public domain by the National Institutes of Health, Bethesda, MD, USA), all images were converted into 8-bit images. To blur the scattering noise, ImageJ plug-in tools of background subtraction and erosion filter (two pixels) were used. Niblack auto local threshold was applied to binarize the images to subdivide the choroid into stromal or luminal segments (Fig 1) [27]. The stromal or luminal area at each sector was calculated with built-in measurement tools in ImageJ.

### Statistical analyses

The baseline characteristics and number of injections of the patients were summarized. For univariate analyses, a linear mixed model was used between changes in BCVA at 12 months (ΔBCVA_12_) and the variables listed herein: baseline BCVA (BCVA_base_), baseline luminal choroidal area (Lumina_base_), baseline stromal choroidal area (Stroma_base_), the change in total choroidal area at 3 months (ΔTotal_3_), the change in luminal choroidal area at 3 months (ΔLumina_3_), the change in stromal choroidal area at 3 months (ΔStroma_3_), the change in total choroidal area at 12 months (ΔTotal_12_), the change in luminal choroidal area at 12 months (ΔLumina_12_), and the change in stromal choroidal area at 12 months (ΔStroma_12_). The linear mixed model is equivalent to ordinary linear regression in the model and describes the relationship between the predictor variables and a single outcome variable. However, standard linear regression analysis assumes that all observations are independent of each other. In the current study, the measurements were nested within subjects and, thus, were dependent of each other. Ignoring this grouping of the measurements would result in the underestimation of standard errors of regression coefficients. The linear mixed model adjusts for the hierarchical structure of the data, thus modelling in a way in which measurements were grouped within subjects.

The relationships between ΔBCVA_12_ and nine variables of age, spherical equivalent, BCVA_base_, Lumina_base_, Stroma_base_, ΔLumina_3_, ΔStroma_3_, ΔLumina_12_, and ΔStroma_12_ were analysed using the linear mixed model. Then, all-subset regression analyses were performed for selecting the best model; that is, the optimal linear model was selected among all possible subsets of the set of potential independent variables counting a total of 512 (= 2^9^) combinations (with nine variables of age, spherical equivalent, BCVA_base_, Lumina_base_, Stroma_base_, ΔLumina_3_, ΔStroma_3_, ΔLumina_12_, and ΔStroma_12_) seeking for a model with the smallest second-order bias-corrected Akaike information criterion (AICc) index value. It is recommended that the model selection method should be used to improve the model fit by removing redundant variables, because the degrees of freedom decrease with a large number of variables in a multivariate regression model [37, 38]. All-subset regression analysis was introduced in the current study because it has some advantages such as relatively low risk of overlooking the best model [39, 40]. The AIC is a well-known statistical measure used in model selection, and the AICc is a corrected version of the AIC, which provides an accurate estimation even when the sample size is small [41].

For comparison, another optimal linear model for ΔBCVA_12_ was identified when only five variables of age, spherical equivalent, BCVA_base_, Total_base_, ΔTotal_3_ and ΔTotal_12_ (2^6^ = 64 combinations) were used, where choroidal thickness was evaluated as the total area. The log likelihood of these optimal models was then compared using AICc difference to determine if using the luminal or stromal area separately or using the choroidal area as a whole would result in a better model.

All statistical analyses were performed with R program (R software version 3.3.2, http://www.r-project.org/). A p value < 0.05 was considered as statistically significant. Multiple comparisons were corrected using the Bonferroni method. P-value of 0.0045 was regarded as statistically significance with correction of the Bonferroni method.

## Results

The baseline characteristics and number of injections of the patients are shown in Table 1. The average number of the injections until 12 months was 7.5±0.5 (range, 7–8). The macula dry percentages at 3 months and 12 months were 100% and 83.3%, respectively. The choroidal areas were divided into luminal and stromal choroid with binarization of the choroid (Fig 1).

**Table 1:** Baseline characteristics and number of injections of the patients.

	Mean ± SD	Range
Age, years	73.8±10	53 to 87
Sex, number (%)	Male: 13 (72%), Female: 5 (28%)	
Spherical Equivalent	- 0.45±2.2	- 5 to 4.25
Baseline BCVA (logMAR)	0.37±0.38	- 0.079 to 1.10
Number of injections	7.5±0.5	7 to 8

BCVA = best-corrected visual acuity; SD = standard deviation; logMAR = logarithm of the minimum angle of resolution.

The average total, luminal, and stromal areas are shown in Table 2. Both the stromal and the luminal areas were decreased at 3 and 12 months compared to baseline (p < 0.0001, p < 0.0001, p < 0.0001 and p < 0.0001, respectively; linear mixed modelling, liner mixed model with Bonferroni method).

**Table 2:** Changes in choroidal area in polypoidal choroidal vasculopathy (PCV) with intravitreal aflibercept injections.

	Baseline (95% Confidence Interval)	3 months (95% Confidence Interval)	P value^a^	12 months (95% Confidence Interval)	P value^b^
Total [10^4^μm^2^]	24.4±9.3 (22.6 to 26.2)	22.5±9.2 (20.8 to 24.3)	< 0.0001	22.0±8.7 (20.4 to 23.7)	< 0.0001
Stroma [10^4^μm^2^]	8.59±2.9 (8.04 to 9.15)	8.17±3.0 (7.59 to 8.75)	< 0.0001	8.09±2.9 (7.54 to 8.63)	< 0.0001
Lumina [10^4^μm^2^]	15.8±6.8 (14.5 to 17.1)	14.4±6.4 (13.1 to 15.6)	< 0.0001	13.9±6.1 (12.8 to 15.1)	< 0.0001

^a^ Difference in values between baseline and 3 months; linear mixed modelling

^b^ Difference in values between baseline and 12 months; linear mixed modelling

Changes in the total, luminal, and stromal areas at each sector are shown in S1, S2 and S3 Tables.

### Univariate analyses

Table 3 shows the results of the univariate analyses between ΔBCVA_12_ and other variables (age, BCVA_base_, Total_base_, Lumina_base_, Stroma_base_, ΔTotal_3_, ΔLumina_3_, ΔStroma_3_, ΔTotal_12_, ΔLumina_12_, or ΔStroma_12_). Background characteristics of age, BCVA_base_, Total_base_, Lumina_base_, and Stroma_base_ were significantly associated with ΔBCVA_12_ (p < 0.00001, p < 0.00001, p < 0.00001, p < 0.00001, p < 0.00001, respectively).

**Table 3:** Univariate analyses between ΔBCVA_12_ and variables in polypoidal choroidal vasculopathy (PCV) with intravitreal aflibercept injections.

	Mean ± SD (95% Confidence Interval)	Value	Standard Error	P Value
Age (years)	73.8 ± 10 (71.9 to 75.7)	0.012	0.0028	< 0.0001
Spherical equivalent	- 0.45 ± 2.2 (- 0.87 to - 0.03)	0.030	0.013	0.0286
BCVA_base_^a^	0.37 ± 0.37 (0.30 to 0.44)	-0.33	0.074	< 0.0001
Total_base_^a^ [μm^2^]	24.4×10^4^ ± 9.3×10^4^ (22.6×10^4^ to 26.2×10^4^)	- 1.8×10^−6^	2.7×10^−7^	< 0.0001
Lumina_base_^a^ [μm^2^]	15.8×10^4^ ± 6.8×10^4^ (14.5×10^4^ to 17.1×10^4^)	- 2.7×10^−6^	3.6×10^−7^	< 0.0001
Stroma_base_^a^ [μm^2^]	8.6×10^4^ ± 2.9×10^4^ (8.0×10^4^ to 9.1×10^4^)	- 4.4×10^−6^	9.5×10^−7^	< 0.0001
ΔTotal_3_^b^ [μm^2^]	- 1.9×10^4^ ± 2.5×10^4^ (- 2.3×10^4^ to - 1.4×10^4^)	0.3×10^−6^	1.2×10^−6^	0.79
ΔLumina_3_^b^ [μm^2^]	- 1.4×10^4^ ± 2.1×10^4^ (- 1.8×10^4^ to - 1.0×10^4^)	0.9×10^−6^	1.4×10^−6^	0.53
ΔStroma_3_^b^ [μm^2^]	- 0.42×10^4^ ± 1.3×10^4^ (- 0.7×10^4^ to - 0.2×10^4^)	- 3.5×10^−6^	2.3×10^−6^	0.13
ΔTotal_12_^c^ [μm^2^]	- 2.4×10^4^ ± 3.3×10^4^ (- 3.0×10^4^ to - 1.7×10^4^)	1.9×10^−6^	0.9×10^−6^	0.036
ΔLumina_12_^c^ [μm^2^]	- 1.9×10^4^ ± 2.6×10^4^ (- 2.4×10^4^ to - 1.3×10^4^)	2.6×10^−6^	1.1×10^−6^	0.024
ΔStroma_12_^c^ [μm^2^]	- 0.51×10^4^ ± 1.5×10^4^ (- 0.8×10^4^ to - 0.2×10^4^)	1.3×10^−6^	2.0×10^−6^	0.52

SD = standard deviation; BCVA = best-corrected visual acuity; Total = total choroid; Lumina = luminal choroid; Stroma = stromal choroid. Linear mixed modelling. P Value of less than 0.0045 was regarded as statistically significant using Bonferroni method. ΔTotal = difference of total choroid; ΔLumina = difference of luminal choroid; ΔStroma = difference of stromal choroid.

^a^Values at baseline.

^b^Changes between baseline and 3 months.

^c^Changes between baseline and 12 months.

### Multivariate analyses using an optimal linear model selection

Table 4 shows the selected variables of the optimal linear model chosen from 2^9^ patterns (= 512) of a combination of seven variables at the baseline and at 3 and 12 months (age, spherical equivalent, BCVA_base_, Lumina_base_, Stroma_base_, ΔLumina_3_, ΔStroma_3_, ΔLumina_12_, and ΔStroma_12_) for ΔBCVA_12_ (Model 1, AICc = -25.4).

**Table 4:** Multivariate analyses between ΔBCVA12 and variables using binarization of the choroid at baseline, 3 and 12 months in polypoidal choroidal vasculopathy (PCV) with intravitreal aflibercept injections.

	Value	Standard Error	P Value
Age	0.010	0.0022	< 0.0001
Spherical equivalent	0.017	0.0098	0.081
BCVA_base_	- 0.30	0.054	< 0.0001
Lumina_base_ [μm^2^]	- 2.2×10^−6^	3.0×10^−7^	< 0.0001

BCVA = best-corrected visual acuity; Lumina = luminal choroid; Stroma = stromal choroid; ΔLumina_3_ = difference of luminal choroid between baseline and 3 months; ΔStroma_3_ = difference of stromal choroid between baseline and 3 months; ΔLumina_12_ = difference of luminal choroid between baseline and 12 months; ΔStroma_12_ = difference of stromal choroid between baseline and 12 months. Linear mixed modelling. Stroma_base_, ΔLumina_3_, ΔStroma_3_, ΔLumina_12_, and ΔStroma_12_ were not selected as associated variables.

Table 5 shows the selected variables of the optimal linear model chosen from 2^6^ (= 64) patterns using a combination of five variables, and using the choroidal area as a whole (age, SE, BCVA_base_, Total_base_, ΔTotal_3_ and ΔTotal_12_) for ΔBCVA_12_ (Model 2, AICc = 21.5).

**Table 5:** Multivariate analyses between ΔBCVA_12_ and variables without binarization of choroid at baseline,3 and 12 months in polypoidal choroidal vasculopathy (PCV) with intravitreal aflibercept injections.

	Value	Standard Error	P Value
Age	0.011	0.0022	< 0.0001
Spherical equivalent	0.016	0.010	0.11
BCVA_base_	- 0.31	0.055	< 0.0001
Total_base_ [μm^2^]	- 1.6×10^−6^	2.2×10^−7^	< 0.0001

BCVA = best-corrected visual acuity; Total_base_ = total luminal choroid; ΔTotal_3_ = difference of total choroid between baseline and 3 months; ΔTotal_12_ = difference of total choroid between baseline and 12 months. Linear mixed modelling. ΔTotal_3_ and ΔTotal_12_ were not selected as associated variables.

The AICc of the optimal model that applied binarization to the choroid (Model 1) was smaller than the model without binarization (Model 2) used to explain ΔBCVA_12_ (-25.4 vs -21.5).

## Discussion

In the current study, we investigated the luminal and stromal areas of the choroid in patients with PCV treated with intravitreal aflibercept, and analysed the relationship between these structures and the VA outcomes. To the best of our knowledge, this is the first report investigating the association of the luminal or stromal area of the choroid separately with VA after intravitreal anti-VEGF therapy. Multivariate analyses were conducted, and an optimal linear model was selected to assess changes in VA at 12 months.

First, in the current analysis, baseline choroidal thickness in total was associated with visual changes at 12 months. The association between choroidal thickness at baseline and visual acuity changes after anti-VEGF treatment in PCV has been discussed in recent years [5, 17–19], even though the results were controversial. We conducted multivariate analyses to investigate each relationship between baseline total choroidal area (Total_base_), the change in choroidal thickness (ΔTotal_3_), or other background factors and the change in VA. The data showed that, after adjustment with baseline choroidal thickness, greater decrease in choroidal thickness was significantly associated with poorer change in VA. Choroidal circulation is important for the retina, because oxygen and nutrition to the outer retina is provided from the choroid [20, 21], to sustain better VA [24, 25]. Furthermore, the choroidal circulation removes metabolic waste and debris, from the outer retina and the RPE cells [22, 23]. The results of the current analysis suggested the importance of the choroid for sustaining visual function.

Recently, binarization of the choroidal structure using EDI-OCT images has been used to assess the choroids of PCV patients [27, 28]. This technique has enabled us to subdivide the choroid into luminal and stromal areas in order to perform a detailed analysis of these structures. It was reported that both luminal and stromal choroidal thicknesses decreased after IAI [27]. The current study showed the comparable results, with a significant decrease of both the stromal and luminal areas significantly decreased at 3 and 12 months compared to baseline. Although a study [28] reported the ratio of luminal/stromal area of the outer choroid divided by the L/S ratio of the inner choroid, we did not perform this assessment because defining the boundary line between the inner and the outer choroid was difficult in the current OCT settings.

Furthermore, in the current study, we analysed the relationships between the luminal or stromal choroid and VA separately using multivariate analyses. Because the responses after anti-VEGF treatment were different between the lumen and the stroma [27, 28], the pathological changes or the responses to the treatment might be independent of each other. However, the previous reports assessed the indices between the luminal area and stromal area. The present study analysed the absolute change in the luminal area or the stromal area separately, because it could present each response more clearly than calculating the relative change (indices) between them. The results showed that greater visual improvement at 12 months was significantly associated with younger age, worse baseline BCVA, and greater baseline luminal area.

It is of note that greater luminal choroidal areas at baseline (Lumina_base_) were correlated with better improvement of BCVA. A larger amount of vasculature (i.e., nutrient supply) at baseline, which was represented by a larger luminal area in the subfoveal choroid, has been reported to be important to gain better VA [24, 25]. Considering that the luminal choroid area tends to decrease after continuous aflibercept therapy, an abundance of vascularity in the choroid might be beneficial in order to secure perfusion reserve capacity for vascular nutrition to maintain visual function.

Of note, the best model using variables after subdividing the choroid into luminal and stromal areas (age, spherical equivalent, BCVA_base_, and Lumina_base_) was superior to that without (age, spherical equivalent, BCVA_base_, and Total_base_). The results suggested that the luminal and stromal choroid changed differently and the luminal area of the choroid could be more useful to predict VA outcomes than total choroidal thickness.

Our results suggested that older age was associated with poorer improvement of BCVA. Our results were similar to previous studies, which indicated that aged patients tended to show poorer VA [4, 42]. The current results also indicated that better baseline BCVA correlated with less improvement of VA at 12 months, which was the same result from previous studies [43, 44].

There are some limitations to the current study. First, the current study was a retrospective investigation. A further prospective study is needed to confirm the effects of older age, a larger luminal choroid, and a smaller stromal choroid on the prognosis of PCV after the IAI treatment. Second, the current study follow-up period was relatively short (12 months). Third, because it is not currently possible to perform such binarization processing automatically, it may be still difficult to apply the findings in the present study to clinical practice. However, binarization processing is an established image-processing technique [29–33], and can be performed with freely accessible image-processing software. Further developments of OCT technology that enables automatic binarization processing is warranted in the near future. Finally, the sample size was small in the current study. Only PCV patients who were treated strictly following the TAE protocol were included in the study. As a result, the number of eyes satisfying the criteria was 18 eyes. Because of the relatively small sample size, independent variables with much weaker correlations could be excluded in the optimal model. However, our analysis showed significant association even in the relatively small sample size, suggesting that there was a strong association between the luminal choroid and the visual outcomes. Still, it would be important to carry out a further study using a larger sample size.

In conclusion, luminal choroid areas at baseline were significantly associated with the VA change at 12 months. The results of the current study suggested that the luminal and choroid might reflect potential visual prognosis and could be useful as new biomarkers for potential visual outcome after intravitreal aflibercept therapy for PCV.

## Supporting information

S1 File The data analysed. (XLSX)

S1 Table Changes in total choroidal area (10^4^μm^2^) in polypoidal choroidal vasculopathy (PCV) with intravitreal aflibercept injections. (DOCX)

S2 Table Changes in stromal choroidal area (10^4^μm^2^) in polypoidal choroidal vasculopathy (PCV) with intravitreal aflibercept injections. (DOCX)

S3 Table Changes in luminal choroidal area (10^4^μm^2^) in polypoidal choroidal vasculopathy (PCV) with intravitreal aflibercept injections. (DOCX)
