# Supplementary material for: Choroidal structure as a biomarker for visual acuity in intravitreal aflibercept therapy for polypoidal choroidal vasculopathy
Source: PLoS One. 2018 May 10;13(5):e0197042. doi: 10.1371/journal.pone.0197042 (PMC5945009; doi:10.1371/journal.pone.0197042)
Supplement: S1 Table — (DOCX) [file pone.0197042.s002.docx]

**Supplementary 1. Changes in total choroidal area in polypoidal choroidal vasculopathy (PCV) with intravitreal aflibercept injections.**

|  | Baseline  (95% Confidence Interval) | 3 months  (95% Confidence Interval) | P value^a^ | 12 months  (95% Confidence Interval) | P value^b^ |
| --- | --- | --- | --- | --- | --- |
| Central: horizontal [10^4^μm^2^] | 25.7 ± 10.5  (20.5 - 30.9) | 23.8 ± 10.0  (18.9 - 28.8) | <0.0001 | 22.6 ± 8.8  (18.2 - 26.9) | <0.0001 |
| Central: vertical [10^4^μm^2^] | 25.4 ± 9.7  (20.6 - 30.2) | 23.8 ± 10.4  (18.6 - 29.0) | <0.0001 | 23.4 ± 10.0  (18.4 - 28.4) | <0.0001 |
| Nasal [10^4^μm^2^] | 23.6 ± 9.9 (18.7 - 28.5) | 21.4 ± 9.2  (16.8 - 25.9) | <0.0001 | 21.2 ± 8.9  (16.8 - 25.6) | <0.0001 |
| Temporal [10^4^μm^2^] | 23.1 ± 8.2  (19.0 - 27.2) | 21.6 ± 7.5  (17.9 - 25.3) | <0.0001 | 20.7 ± 7.0  (17.2 - 24.2) | <0.0001 |
| Superior [10^4^μm^2^] | 25.5 ± 8.6  (21.2 - 29.7) | 23.5 ± 9.4  (18.8 - 28.1) | <0.0001 | 23.8 ± 9.0  (19.3 - 28.3) | <0.0001 |
| Inferior [10^4^μm^2^] | 23.0 ± 9.7  (18.2 - 27.8) | 21.0 ± 9.4  (16.4 - 25.7) | <0.0001 | 20.5 ± 8.7  (16.2 - 24.8) | <0.0001 |

^a^Difference in values between baseline and 3 months; Linear mixed modeling

^b^Difference in values between baseline and 12 months; Linear mixed modeling
