# Supplementary material for: Choroidal structure as a biomarker for visual acuity in intravitreal aflibercept therapy for polypoidal choroidal vasculopathy
Source: PLoS One. 2018 May 10;13(5):e0197042. doi: 10.1371/journal.pone.0197042 (PMC5945009; doi:10.1371/journal.pone.0197042)
Supplement: S2 Table — (DOCX) [file pone.0197042.s003.docx]

**Supplementary 2. Changes in stromal choroidal area in polypoidal choroidal vasculopathy (PCV) with intravitreal aflibercept injections.**

|  | Baseline  (95% Confidence Interval) | 3 months  (95% Confidence Interval) | P value^a^ | 12 months  (95% Confidence Interval) | P value^b^ |
| --- | --- | --- | --- | --- | --- |
| Central: horizontal [10^4^μm^2^] | 9.16 ± 3.0  (7.66 - 10.7) | 8.84 ± 3.2  (7.26 - 10.4) | <0.0001 | 8.38 ± 3.0  (6.87 - 9.88) | <0.0001 |
| Central: vertical [10^4^μm^2^] | 9.53 ± 3.0  (8.05 - 11.0) | 9.21 ± 3.4  (7.50 - 10.9) | <0.0001 | 9.06 ± 3.5  (7.33 - 10.8) | <0.0001 |
| Nasal [10^4^μm^2^] | 8.14 ± 3.4  (6.46 - 9.82) | 7.55 ± 3.1  (6.00 - 9.11) | <0.0001 | 7.62 ± 3.0  (6.13 - 9.12) | <0.0001 |
| Temporal [10^4^μm^2^] | 8.09 ± 2.6  (6.82 - 9.36) | 7.55 ± 2.2  (6.48 - 8.62) | <0.0001 | 7.57 ± 2.2  (6.49 - 8.65) | 0.0002 |
| Superior [10^4^μm^2^] | 8.78 ± 2.3  (7.65 - 9.92) | 8.40 ± 2.9  (6.95 - 9.85) | <0.0001 | 8.45 ± 2.8  (7.06 - 9.84) | <0.0001 |
| Inferior [10^4^μm^2^] | 7.85 ± 3.1  (6.31 - 9.40) | 7.47 ± 3.2  (5.86 - 9.07) | <0.0001 | 7.44 ± 2.7  (6.11 - 8.77) | <0.0001 |

^a^Difference in values between baseline and 3 months; Linear mixed modeling

^b^Difference in values between baseline and 12 months; Linear mixed modeling
