# Supplementary material for: Choroidal structure as a biomarker for visual acuity in intravitreal aflibercept therapy for polypoidal choroidal vasculopathy
Source: PLoS One. 2018 May 10;13(5):e0197042. doi: 10.1371/journal.pone.0197042 (PMC5945009; doi:10.1371/journal.pone.0197042)
Supplement: S3 Table — (DOCX) [file pone.0197042.s004.docx]

**Supplementary 3. Changes in luminal choroidal area in polypoidal choroidal vasculopathy (PCV) with intravitreal aflibercept injections.**

|  | Baseline  (95% Confidence Interval) | 3 months  (95% Confidence Interval) | P value^a^ | 12 months  (95% Confidence Interval) | P value^b^ |
| --- | --- | --- | --- | --- | --- |
| Central: horizontal [10^4^μm^2^] | 16.6 ± 7.8  (12.7 - 20.4) | 15.0 ± 7.1  (11.5 - 18.5) | <0.0001 | 14.2 ± 5.9  (11.3 - 17.1) | <0.0001 |
| Central: vertical [10^4^μm^2^] | 15.9 ± 7.1  (12.4 - 19.4) | 14.6 ± 7.1  (11.1 - 18.1) | <0.0001 | 14.4 ± 6.7  (11.0 - 17.7) | <0.0001 |
| Nasal [10^4^μm^2^] | 15.5 ± 7.3  (11.8 - 19.1) | 13.8 ± 6.3  (10.6 - 17.0) | <0.0001 | 13.6 ± 6.3  (10.4 - 16.7) | <0.0001 |
| Temporal [10^4^μm^2^] | 15.0 ± 5.9  (12.1 - 18.0) | 14.1 ± 5.5  (11.3 - 16.8) | <0.0001 | 13.2 ± 5.2  (10.6 - 15.7) | <0.0001 |
| Superior [10^4^μm^2^] | 16.7 ± 6.5  (13.5 - 19.9) | 15.1 ± 6.7  (11.7 - 18.4) | <0.0001 | 15.3 ± 6.5  (12.1 - 18.6) | <0.0001 |
| Inferior [10^4^μm^2^] | 15.2 ± 6.8  (11.8 - 18.5) | 13.6 ± 6.4  (10.4 - 16.8) | <0.0001 | 13.1 ± 6.2  (9.97 - 16.2) | <0.0001 |

^a^Difference in values between baseline and 3 months; Linear mixed modeling

^b^Difference in values between baseline and 12 months; Linear mixed modeling
